# Dynamic Network-Level Traffic Speed and Signal Control in Connected Vehicle Environment

**DOI:** 10.3390/s24206597

**Published:** 2024-10-13

**Authors:** Zihao Yuan, Xiaoqing Zeng

**Affiliations:** The Key Laboratory of Road and Traffic Engineering, Ministry of Education, Tongji University, Shanghai 201804, China; yuanzihao@tongji.edu.cn

**Keywords:** connected vehicles, traffic signal optimization, fuel consumption reduction, emission minimization

## Abstract

The advent of connected vehicles holds significant promise for enhancing existing traffic signal and vehicle speed control methods. Despite this potential, there has been a lack of concerted efforts to address issues related to vehicle fuel consumption and emissions during travel across multiple intersections controlled by traffic signals. To bridge this gap, this research introduces a novel technique aimed at optimizing both traffic signals and vehicle speeds within transportation networks. This approach is designed to contribute to the improvement of transportation networks by simultaneously addressing issues related to fuel consumption and pollutant emissions. Simulation results vividly illustrate the pronounced the effectiveness of the proposed traffic signal and vehicle speed control methods of alleviating vehicle delay, reducing stops, lowering fuel consumption, and minimizing CO_2_ emissions. Notably, these benefits are particularly prominent in scenarios characterized by moderate traffic density, emphasizing the versatility and positive impact of the method across varied traffic conditions.

## 1. Introduction

**Figure 1:**
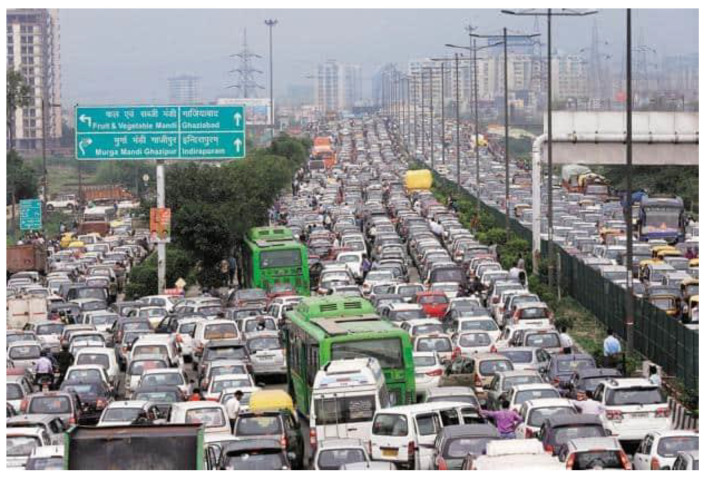
The problem of traffic congestion.

The escalating demand for traffic services poses a growing challenge in attaining optimal service levels through conventional control systems and existing traffic infrastructure. In the year 2022, global commuters faced considerable challenges, with a notable 15% increase in traffic-related issues over the past five years. This resulted in a significant waste of resources, with commuters globally wasting an astonishing 3.2 billion gallons of fuel and 7.4 billion hours due to traffic delays [1]. Figure 1 depicts the problem of traffic congestion.

Based on the most recent data available as of 2022, an overwhelming majority, approximately 97%, of global traffic signals rely on historical traffic patterns as the foundational basis for signal timing control [2]. This traditional approach exhibits reduced adaptability to sudden shifts in real-time traffic demand and dynamic conditions. In contrast, within the remaining 3% of intersections, actuated signals present an alternative approach by utilizing fixed-point vehicle detection positioned upstream of the intersection [3].Despite the integration of actuated signals, this localized detection method has inherent limitations, unable to capture the dynamic variability intrinsic to traffic flow [4].

To further elaborate, localized detection methods, such as fixed-point vehicle detection, face challenges in effectively accommodating the diverse and evolving nature of traffic conditions. These challenges arise from the inability of fixed-point detection to account for sudden changes in traffic patterns and unexpected congestion events. Consequently, the limitations in capturing dynamic traffic variations impede the overall efficacy of adaptive traffic signal control systems that incorporate fixed-point detection mechanisms [5]. The efficacy of these systems becomes contingent on the precision and real-time responsiveness of the employed vehicle detection mechanisms.

In light of these limitations, it becomes imperative to explore alternative technologies and methodologies for optimizing signal control in the continuously evolving landscape of urban mobility. The pursuit of innovative solutions is crucial to overcome the challenges posed by the limitations inherent in traditional and localized signal control approaches.

**Figure 2:**
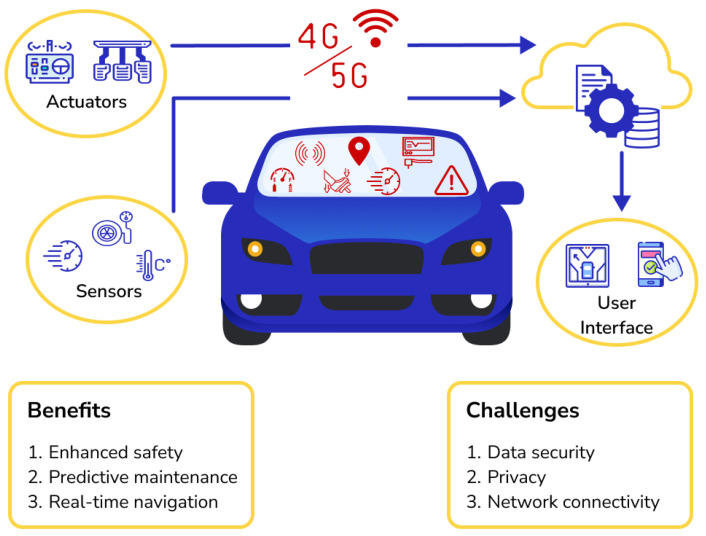
Vehicle data collection through connected vehicle technology.

In contrast, connected vehicle technology plays a pivotal role by enabling intelligent signal controllers to monitor real-time traffic conditions at intersections. This is achieved through the collection of precise location, speed, and acceleration data from approaching connected vehicles, ultimately preventing congestion and enhancing the overall efficiency of signalized intersections [6]. Figure 2 depicts vehicle data collection through connected vehicle technology.

On the other hand, relevant researches have shown another problem in the urban traffic signal control field. Recent research indicates that suboptimal traffic signal control contributes to 5 to 10 percent of total commuter delays worldwide [7]. Conventional signal control strategies focused on mobility objectives often lead to excessive fuel consumption through the creation of stop-and-go traffic patterns [8]. Additionally, studies have shown that that ill-advised vehicle maneuvers, such as sudden acceleration, abrupt braking, and prolonged idling, can markedly escalate both fuel consumption and emissions [9]. For example, instances of aggressive driving, like rapid acceleration from a standstill or abrupt stops, have been identified as key contributors to heightened fuel usage and pollutant releases, exacerbating the environmental impact of vehicular activities. Therefore, appropriately changing the vehicle speed to minimize sharp acceleration, deceleration and stopping at signalized intersections is imperative to reducing fuel consumption and emissions [10].

**Figure 3:**
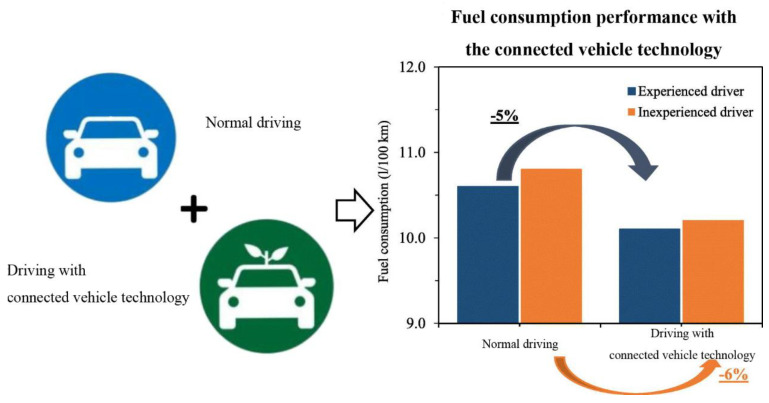
Vehicle fuel consumption reduction through connected vehicle technology.

Given the transformative potential of connected vehicle technology in optimizing traffic dynamics and fuel consumption, there is an imperative to advance signal control and vehicle speed control systems. These systems should leverage the latest developments in connected vehicle technology to address the challenges associated with traditional signal control methods and enhance overall traffic management efficiency. Figure 3 depicts vehicle fuel consumption reduction through connected vehicle technology.

The increasing demand for traffic services has made it difficult for conventional control systems and existing infrastructure to maintain optimal service levels. In 2022, traffic-related issues rose by 15%, leading to significant fuel and time wastage. Most global traffic signals (97%) rely on outdated, historical traffic patterns, which limits their adaptability to real-time changes. While some intersections use actuated signals with fixed-point vehicle detection, this method struggles to accommodate dynamic traffic conditions. In contrast, connected vehicle technology offers a solution by collecting real-time data, such as vehicle location, speed, and acceleration, to optimize signal control, reduce congestion, and minimize fuel consumption. Advancing signal and speed control systems with connected vehicle technology could significantly enhance traffic efficiency and environmental impact.

First, the Introduction provides the research background and objectives, emphasizing the limitations of traditional traffic signal control and the necessity of optimizing traffic using connected vehicle technology. The Literature Review summarizes existing studies in the field, analyzing both traditional and intelligent traffic control methods and their application in modern traffic environments. The Research Methodology section presents an innovative traffic signal and vehicle speed control algorithm integrating a metaheuristic optimization approach and is validated through a microscopic simulation model. The Case Study section validates the effectiveness of the proposed algorithm through simulation tests demonstrating its performance in optimizing traffic signals and vehicle speed under different traffic conditions. Finally, the Conclusion summarizes the research findings, highlighting the benefits of combining signal control with vehicle speed control and proposes future research directions such as real-world validation and integration with autonomous driving technologies

## 2. Literature Review

The rapid expansion of urbanization, improvements in economic conditions, and the availability of affordable and convenient transportation options have collectively contributed to a significant increase in car usage across numerous cities worldwide. In the United States, vehicle miles traveled (VMT) increased by 167% from 1970 to 2022, reflecting a notable upward trend in car usage [11]. This growing reliance on cars presents major challenges to transportation sustainability, particularly in the form of worsening traffic congestion and pollution. To address these challenges, urban traffic control methods have continuously evolved and innovated to accommodate increasing traffic demands [12]. Intersection design plays a critical role in managing the right-of-way for vehicles approaching from multiple directions, thereby improving the overall efficiency of traffic systems [13].

Currently, most traffic signal control systems rely on infrastructure-based sensors such as loop detectors, video detectors, and radar to collect traffic data and make decisions based on simple logic [14]. However, these detectors have inherent limitations, as they can only capture a singular snapshot of a vehicle’s data when it passes through the detection area, lacking continuous geographic information such as speed, acceleration, and position [15]. This limitation severely hampers their ability to provide comprehensive insights into dynamic traffic conditions, making it difficult for traditional systems to meet the complexities of modern urban mobility.

Moreover, these detection systems come with significant installation and maintenance costs, adding to the overall financial burden of implementing and maintaining traffic signal control infrastructure [16]. In the event of detector malfunctions, the efficiency of existing signal systems is further reduced, exacerbating traffic problems [17]. As a result, there is an urgent need for more robust and resilient traffic control systems in real-world applications.

The emergence of intelligent transportation systems (ITS) has introduced numerous innovative applications, with connected vehicle technology becoming a key driver of these advancements. Connected vehicle technology establishes wireless communication links between smart vehicles and roadside infrastructure [18,19,20]. In a connected vehicle environment, signal systems play a critical role in facilitating communication between vehicles and infrastructure [21]. This communication occurs through various channels, including vehicle-to-vehicle (V2V), vehicle-to-infrastructure (V2I), and infrastructure-to-vehicle (I2V), enabling high-speed information exchange [22]. The technological foundations of these signal systems provide a strong framework for efficient information sharing and coordination [23].

In practical terms, connected vehicle technology allows for the real-time adjustments of signal timings based on traffic flow data from vehicles, minimizing unnecessary delays [24]. Additionally, the integration of connected vehicles brings a range of benefits, such as crash reduction, travel time optimization, and energy efficiency improvements [25]. By incorporating connected vehicle technologies into urban traffic signal control and speed control systems, these benefits can be fully realized, advancing overall traffic management. As a result, signal timings can dynamically adjust based on actual traffic flow, reducing unnecessary stops and delays [26]. Furthermore, by analyzing historical and real-time vehicle data, traffic management systems can predict traffic patterns and adjust signal timings proactively, helping to prevent congestion and improve the overall efficiency of signalized intersections [27]. Additionally, connected vehicles can send requests or feedback to influence signal timings, allowing the system to prioritize certain traffic movements based on real-time demand, optimizing the overall traffic flow [28].

In the Internet of Vehicles (IoV) environment, speed control is a critical issue for ensuring coordinated operations, traffic safety, and efficient driving. Traditional speed control methods rely on a vehicle’s autonomous perception and feedback systems. However, in the IoV environment, vehicles can share real-time environmental and traffic data through network interconnection, providing new opportunities and challenges for speed control [29]. Through V2V communication, vehicles can obtain speed and position data from nearby vehicles, allowing them to adjust their speed to avoid collisions and optimize following distances, significantly improving traffic flow stability and safety [30].

V2I technology enables vehicles to receive traffic signals, road conditions, and speed limit information from roadside infrastructure [31]. This infrastructure-based control method provides vehicles with more comprehensive traffic information, allowing for speed control optimization not only at the individual vehicle level but also across the entire traffic network, reducing congestion and improving energy efficiency [32].

With the development of artificial intelligence, more intelligent algorithms have been applied to speed control in IoV. These methods include reinforcement learning, fuzzy control, and genetic algorithms, which enable vehicles to adaptively adjust their speed according to dynamic traffic environments [33]. In recent years, researchers have also proposed deep learning-based speed prediction models, which can forecast future traffic trends based on historical and real-time data, providing more forward-looking suggestions for speed control [34]. The “Greenwave” is a traffic signal coordination control technique aimed at adjusting the timing of a series of traffic signals along a street, allowing vehicles to encounter green lights continuously at an appropriate speed, reducing the number of stops and waiting times [35]. This method helps improve traffic flow, reduce congestion, and lower vehicle fuel consumption and emissions. By setting a specific “Greenwave band,” vehicles can smoothly pass through multiple consecutive green lights within a certain speed range after entering a specific road section [36].

## 3. Research Methodology

Within this chapter, we present an innovative traffic signal control algorithm designed to enhance the optimization of critical signal timing parameters, specifically, traffic signal timing and signal offset, across the entirety of an urban street network. The proposed algorithm incorporates a scalable solution technique, providing a versatile approach to effectively address and resolve the identified optimization challenge. To illustrate the applicability and efficacy of the algorithm, we apply it to a case study network, considering diverse traffic volume patterns to assess its performance under varying conditions. This approach ensures a comprehensive evaluation of the algorithm’s adaptability and effectiveness in real-world scenarios.

The proposed traffic signal control algorithm was designed with two main objectives: optimizing signal timing parameters, such as cycle length, phase split, and signal offset, across multiple intersections in an urban street network. The following steps outline the method in detail:

(a) Problem Formulation:

The optimization problem is framed as a multi-objective problem, where the goal is to minimize total vehicle delay and queue length at signalized intersections while ensuring smooth traffic flow. The objective functions are defined in terms of average vehicle delay at intersections and travel time reliability across the entire network.

(b) Optimization Variables:

Signal Timing Parameters: These include the cycle length, which refers to the total duration of one complete signal cycle, and the phase split, which determines how much green time is allocated to each movement during the cycle. Signal Offset: The offset is a time delay introduced between consecutive intersections to ensure better progression for vehicles moving along a corridor.

(c) Scalable Solution Technique:

The algorithm employs a metaheuristic optimization approach, such as a genetic algorithm (GA) or particle swarm optimization (PSO), chosen for their adaptability in handling large, complex networks. The scalability of the solution is maintained by dividing the network into manageable sub-networks and optimizing each sub-network independently, followed by a final integration step to ensure network-wide coordination.

(d) Modeling the Network:

A microscopic simulation model (e.g., VISSIM or SUMO) was integrated with the optimization algorithm to simulate real-world traffic conditions. The model captures the behavior of vehicles, including acceleration, deceleration, and interaction at intersections, providing a realistic assessment of the performance under varying traffic volumes and patterns.

(e) Performance Metrics:

The algorithm was evaluated using performance metrics such as average vehicle delay, number of stops per vehicle, and queue length. Additionally, fuel consumption and emission levels may also be considered to assess the environmental impact.

(f) Case Study Application:

The algorithm was applied to a real-world network, such as a section of an urban arterial or an entire city district. Diverse traffic volume patterns were introduced into the model to simulate different demand scenarios, including peak-hour traffic and off-peak conditions. The adaptability of the algorithm was assessed by examining its ability to maintain optimal performance across these scenarios.

(g) Solution Process:

Initialization: The algorithm begins with an initial feasible solution, which is typically derived from historical data or a pre-defined baseline signal plan. Iteration and Convergence: Through an iterative process, the algorithm adjusts the signal timing and offsets, improving upon the initial solution until a stopping criterion is met (e.g., a maximum number of iterations or convergence to a minimal delay).

(h) Validation:

The final solution was validated against real-world traffic data to ensure its robustness and reliability. The simulation results were compared with field data, if available, to confirm the improvement in traffic performance.

### 3.1. Signal Control Algorithm

#### 3.1.1. The Objective Function at the Intersection Level

At the intersection level, the primary goal of the signal control algorithm is to minimize the travel delay experienced by vehicles at each intersection. This involves formulating an objective function that quantifies the total delay. The algorithm then determines the signal timing for each direction, aiming to identify the configuration that minimizes the established objective function. This approach ensures that the chosen signal timings are optimized to enhance traffic flow efficiency and reduce overall delays at the intersection.

The objective function is defined by Equation (1).

(1) $$MinD\left(g\right)=\sum_{m=1}^{N}D_{m}\left(g\right)$$

Table 1 provides a comprehensive overview of the key notations, along with their corresponding definitions and detailed descriptions.

The formula of $D_{m}\left(g\right)$ is defined by Equation (2).

(2) $$D_{m}\left(g\right)=T_{m}\left(g\right)-A_{m}\left(g\right)$$

where the travel delay $D_{m}\left(g\right)$ of each vehicle is equal to be the actual departure time of that vehicle leaving the stop bar, $T_{m}\left(g\right)$, minus the virtual departure time of the same approaching vehicle with free flow speed, $A_{m}\left(g\right)$.

#### 3.1.2. The Control Constraints at Intersection Level

At the intersection level, the control constraint of the signal control algorithm is defined by Equation (3).

(3) g≥5 s

The constraint of the green time of each signal phase in each intersection needs a control constraint, in order to make sure that all queueing vehicles can pass the intersection within the green signal period.

#### 3.1.3. The Objective Function at the Network Level

At the network level, the signal control algorithm aims to optimize the performance across multiple intersections by formulating an objective function. In this context, the primary goal is to maximize the bandwidth of the arterial road network. The algorithm evaluates various signal offset configurations for each intersection, seeking to identify the optimal signal offset that maximizes the established objective function. This approach ensures that the signal offsets are strategically adjusted to enhance overall network capacity and promote efficient traffic flow along the arterial road.

The objective function is defined by Equation (4).

(4) Max b

**Figure 4:**
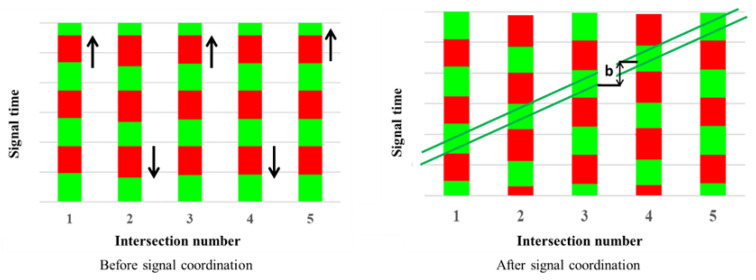
An example of the GreenWave bandwidth.

The bandwidth for one direction is a portion of time that the synchronized green wave lasts in a cycle length. A simple example of the GreenWave bandwidth is shown by Figure 4. Red represents a red light (stop signal), green represents a green light (go signal), The arrows indicate the direction of traffic flow or the trend of signal changes, Upward arrows represent the forward sequence or increasing trend of signal phases, Downward arrows represent the descending or reversing trend of signal phases. “b” indicates the time offset or phase difference between signals.

#### 3.1.4. Control Constraints at the Network Level

At the network level, the signal control algorithm mandates a uniform cycle length for all intersections, set equal to the maximum value among the cycle lengths of all intersections. This strategic synchronization ensures optimal coordination across the traffic network. In the proposed algorithm, the concept of the GreenWave bandwidth represents the temporal span during which vehicles can traverse the arterial road without encountering red signals. It is crucial to acknowledge that the synchronization of signal offsets contributes to maximizing this GreenWave bandwidth, aligning with the fundamental principles of coordinated signal control.

Firstly, physical constraints govern the generation of signal offsets for each intersection within the prescribed GreenWave bandwidth. Such primary constraint stems from the consideration that the length of the GreenWave bandwidth must not exceed the duration of the green light in the arterial road direction at each intersection. This constraint is inherently tied to the operational dynamics of traffic signal control. For both directions of the arterial road, the constraints on green time and bandwidth are defined by Equations (5) and (6), emphasizing the precision and rigor applied in designing signal control algorithms within the context of optimizing the GreenWave bandwidth.

(5) $$w_{i}+b\leq C-r_{i},\;w_{i}>0$$

(6) $$\bar{w_{i}}+\bar{b}\leq C-\bar{r_{i}},\;\bar{w_{i}}>0$$

where C represents the cycle length of the traffic signal, while $w_{i}$ signifies the time interval between the end of red time and the commencement of the bandwidth on the inbound direction. Similarly, $\bar{w_{i}}$ denotes the time interval between the end of red time and the initiation of bandwidth on the outbound direction. The variable *b* is designated as the bandwidth variable, and $r_{i}$ represents the red time on the inbound direction, with $\bar{r_{i}}$ representing the red time on the outbound direction.

Secondly, for the two directions of inbound and outbound, usually the bandwidths for the two directions of the arterial road are set equal, which is defined by Equation (7).

(7) $$b=\bar{b}$$

**Figure 5:**
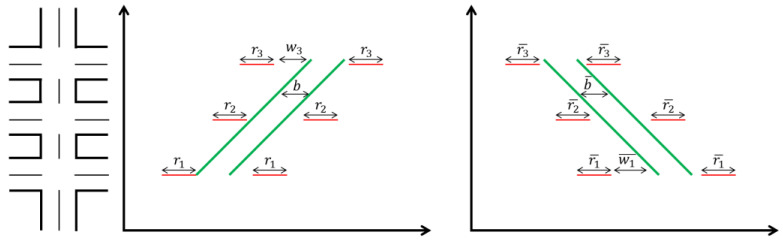
Bandwidth constraints of each intersection.

A simple example of Equations (5) and (6) is shown by Figure 5. This example is shown as an illustration of a three-intersection arterial network. The red solid lines indicate the red signal. The green band indicates the green wave.

The primary distinction of this study compared to conventional methods lies in its innovative approach to optimizing the GreenWave bandwidth while allowing for the use of varying cycle lengths across intersections within a synchronized signal control framework. While signal coordination is widely accepted and typically requires a common cycle length, as clearly outlined in traffic engineering textbooks and signal timing manuals, this study explores the potential of optimizing traffic flow even when intersections operate with different cycle lengths. This approach challenges the conventional assumption that uniform cycle lengths are necessary for effective coordination. Instead, by synchronizing signal phases across the network while maintaining flexible cycle lengths, the system can dynamically adjust to varying traffic demands, allowing vehicles to traverse multiple intersections on arterial roads with minimal stops. This method provides greater adaptability in managing complex traffic patterns, offering a novel solution to improve traffic flow where uniform cycle lengths may not be feasible.

Additionally, the proposed algorithm introduces a more detailed and rigorous approach to generating signal offsets. It accounts for physical constraints that ensure the GreenWave bandwidth does not exceed the green light duration in either inbound or outbound directions. The use of Equations (5) and (6) underscores the precision required in balancing the green and red times at each intersection, which is often neglected in simpler methods that may only focus on a single intersection or assume symmetric traffic conditions. The synchronization of signal offsets based on both directions of traffic flow is another unique feature of this approach, ensuring that vehicles moving in either direction along the arterial road benefit from GreenWave.

Finally, the assumption that bandwidths in both directions of the arterial road are equal (Equation (7)) differentiates this approach from methods that might prioritize one direction over the other, potentially neglecting optimization for both inbound and outbound traffic.

### 3.2. Vehicle Speed Control Algorithm in a Partial-Connected Vehicle Environment

#### 3.2.1. Queue Estimation at Intersections

This study focused on defining queuing vehicles as those that approach an intersection, decelerate to a full stop, and await the green light to proceed. Connected vehicles play a pivotal role by transmitting vital information to the roadside unit, including vehicle ID, location, queue time, and speed. The research adopts a Poisson distribution to model vehicle arrivals, resulting in a queue with an associated arrival rate. This statistical approach provides a realistic representation of the stochastic nature of traffic flow. It is worth emphasizing that the study’s focus on a single-lane context and the quantification of connected vehicles contributes to a more streamlined and analytically manageable investigation, enabling a thorough understanding of the queuing dynamics at signalized intersections.

**Figure 6:**
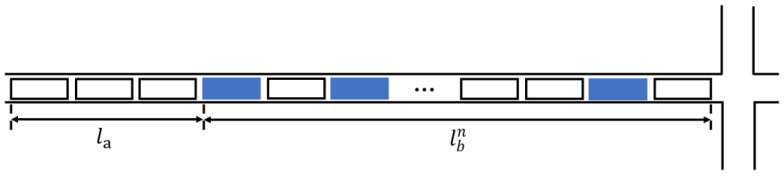
Diagram of *n* connected vehicles.

Figure 6 shows the diagram of queue estimation when there are *n* connected vehicles. The solid rectangle represents a connected vehicle, and *n* denotes the total number of connected vehicles present. The connected vehicle penetration rate is determined by calculating the proportion of connected vehicles to the overall vehicle count. This metric provides insights into the extent of integration and adoption of connected technology within the vehicular network. $r_{s}$ represents the start time of the red light, $r_{e}$ represents the end time of the red light, when there is only one connected vehicle, the speed of the shockwave ($v_{1}$) can be determined as follows:

(8) $$v_{1}=\frac{l_{b}}{t_{1}-r_{s}}$$

where $t_{1}$ is the stop time of this connected vehicle, $l_{b}$ signifies the queue length in front of the connected vehicle, which is dictated by the distance between the connected vehicle and the stop line positioned at the intersection.

Therefore, when there are only *n* connected vehicles, we can assume that the speed of the shockwave is determined as follows:

(9) $$v_{n}=\frac{1}{n-1}\sum_{i=1}^{n-1}\frac{l_{b}^{n}-l_{b}^{i}}{t_{n}-t_{i}}\;(i=1,\;2,\;\ldots ,\;n-1)$$

where *i* is the connected vehicle, $l_{b}^{n}$ is the queue length of the last connected vehicle, $l_{b}^{i}$ is the queue length before the *i*th connected vehicle. The calculation of this length is based on the difference between the position of the last connected vehicle and the stop line at the intersection. $t_{n}$ is the stop time of the nth connected vehicle, and $t_{i}$ is the stop time of the *i*th connected vehicle. For $l_{b}^{n}$, the blue box represents the queue length of the last connected vehicle, i.e., the distance from the stop line to the last vehicle in the queue.

The queue length of unconnected vehicles after the *n*th connected vehicle ($l_{a})$ is determined as follows:

(10) $$l_{a}=v_{n}(r_{e}-t_{n})$$

The total queue length can be calculated by Equation (11):

(11) $$l_{i}=l_{a}+l_{b}^{n}=v_{n}(r_{e}-t_{n})+l_{b}^{n}$$

(1) Signal Control Responds to Dynamic Changes in Queue Prediction

The core of queue prediction and signal control lies in how to dynamically adjust signal timing according to queue length and traffic flow to ensure smooth traffic. Although the current document discusses queue prediction models, there is a lack of detailed explanation on how the signal control system responds to these predictions. Here are the points that need to be added:

- Suppose the queue length at a particular intersection exceeds 10 vehicles during peak hours. Based on the prediction model, the signal control system could extend the green light duration for the current cycle until the queue length reduces to a reasonable range (e.g., five vehicles). During this adjustment, the signal system needs to continuously assess the change in queue length and revert to the normal green light duration in the next cycle.
- In lower traffic scenarios, the signal control system could reduce the green light time based on shorter queue lengths, thus minimizing idle time and optimizing the overall traffic flow.
- If the traffic flow in the east–west direction suddenly increases, the system can reduce the green light time for the north–south direction and allocate more time to the east–west direction to quickly alleviate queues and prevent prolonged congestion.
- If the queue length at an intersection exceeds the normal range, the system can automatically adjust the signal offsets at neighboring intersections, allowing traffic at the congested intersection to pass first, reducing the likelihood of further congestion. This dynamic adjustment of signal offsets is based on real-time feedback from queue length predictions.

(2) Inter-Cycle Control Mechanism

Current queue prediction models are based on queue lengths and traffic conditions within a single cycle. However, queue changes in real traffic may span multiple cycles. The document should detail how to manage queues across cycles, that is, how the signal control system continuously optimizes signal timing over multiple cycles to effectively manage prolonged queues.

- Suppose the queue length at an intersection reaches 20 vehicles, and a single green light cycle cannot clear all vehicles. The system could plan to extend the green light time over the next three cycles, increasing green light time by 5 s each cycle until the queue length returns to normal. In this way, the system ensures smooth traffic flow over multiple cycles without causing excessive delays
- If the system predicts continuous queue growth over the next two cycles, it can preemptively adjust the signal offset or green light duration for the next cycle, ensuring that vehicles can pass more quickly in future cycles and preventing more severe congestion

(3) Using Predictive Feedback for Dynamic Adjustments

It is important to emphasize that the queue prediction model should not only be used for current cycle adjustments but also serve as a basis for optimizing signal timing in future cycles. The document can include discussions on how to use real-time feedback mechanisms in combination with predictive results to dynamically optimize signal timing for future cycles.

- If the actual traffic flow at an intersection in the current cycle is 30% higher than the prediction model, the system can use that feedback to adjust the green light duration for the next cycle, releasing more vehicles early and preventing future congestion from worsening.

#### 3.2.2. Speed Control Method for Single-Signalized Intersections

At the individual intersection level, the core of the vehicle speed control strategy lies in ensuring that when a vehicle approaches the end of the queue formed during a red-light phase, the shockwave precisely propagates to the queue’s end. This mechanism is designed to optimize traffic flow dynamics by synchronizing the movement of the last vehicle in the queue with the arrival of the shockwave, thereby enhancing the efficiency of vehicle movement through signalized intersections. Therefore, the optimal speed can be calculated as follows:

(12) $$v=\frac{L_{i}-l_{i}}{g_{ij}+\frac{l_{i}}{v_{i}}}$$

In the provided equation, the numerator represents the difference between the distance from the vehicle to the stop line ($L_{i}$) and the road space taken up by the queueing vehicles ($l_{i}$), estimated using Equation (11). The denominator in this context signifies the time duration after the green light starts, by the moment when the last queueing vehicle starts to move. This formulation reflects the influence of queue length on the optimal speed calculation, a key aspect of adaptive traffic speed control.

Transitioning to the model for speed control at single signalized intersections, the recommended driving speed for a vehicle after control must adhere to the following criteria:

(13) $$v=\left\{\begin{array}{c} \frac{L_{i}-l_{i}}{g_{ij}+\frac{l_{i}}{v_{i}}}\;,\;\;v_{min}\leq v\leq v_{max}\;\\ v_{max}\;,\;\;v>v_{max}\\ v_{min}\;,\;\;v<v_{min} \end{array}\right.$$

Equation (13) highlights the necessity of restricting the recommended speed within predefined limits, specifically $v_{max}$ and $v_{min}$. This constraint on speed aligns with established traffic safety principles, recognizing the importance of maintaining speeds within a specified range to ensure safe and efficient vehicle movement. Moreover, it is noteworthy to consider the broader context of speed limits in traffic engineering. Speed limits are often set based on factors such as road type, surrounding environment, and pedestrian activity, emphasizing the multifaceted nature of speed regulation. Equation (13) reflects the integration of these principles into the specific context of optimizing vehicle speeds at signalized intersections, contributing to a comprehensive and adaptive approach to traffic control.

#### 3.2.3. Speed Control Method for Multiple Signalized Intersections

The essential aspect of speed control is to guarantee that as a vehicle reaches the end of the queue, induced by the red light, the shockwave is precisely transmitted to the end of the queue.

Drawing inspiration from this conceptual framework, we make the assumption that, at any given time, the distance between the vehicle and the following intersections is already known and represented by $L_{i}$ (i=1,2,3…N). Here, the subscript *i* signifies the intersection number while connected vehicles are travelling along the arterial road.

To enhance the dynamic utilization of traffic signal status for calculating the optimal speed of connected vehicles, we introduced variables $g_{ij}$ and $r_{ij}$. The former represents the start time of the *j*th green time of the *i*th intersection. The latter denotes the start time of the *j*th red time of the *i*th intersection, respectively. This strategic incorporation aligns with the broader field of traffic signal control optimization, where data transmission of the signal timing and the connected vehicle is essential for adaptive traffic management. Building on this, the formulation of the feasible speed set for a vehicle intending to traverse the *i*th intersection is expressed as follows:

(14) $$\left[\frac{L_{i}}{r_{ij}},\frac{L_{i}-l_{i}}{g_{ij}+\frac{l_{i}}{v_{i}}}]\cap [v_{min},v_{max}\right]$$

where $v_{min}$ denotes the minimum speed limit and $v_{max}$ represents the maximum speed limit respectively. Incorporating additional knowledge related to traffic dynamics, it is noteworthy that setting speed limits is a crucial aspect of traffic management, ensuring both safety and efficiency. $l_{i}$ represents the estimated length of the queue before the *i*th signalized intersection, a parameter critical for assessing congestion levels and optimizing traffic flow, which is estimated by Equation (11); $v_{i}$ is the speed of the shockwave before the *i*th signalized intersection, which is estimated by Equation (9). If

(15) $$\left[\frac{L_{i}}{r_{ij}},\frac{L_{i}-l_{i}}{g_{ij}+\frac{l_{i}}{v_{i}}}\right]\cap \left[\frac{L_{i+1}}{r_{i+1j}},\frac{L_{i+1}-l_{i+1}}{g_{i+1j}+\frac{l_{i+1}}{v_{i+1}}}\right]\cap \left[v_{min},v_{max}\right]=\left[v_{min}^{\prime },v_{max}^{\prime }\right]\neq \emptyset$$

then the vehicle can pass through the *i*th intersection and *i +* 1 th intersection within the speed range of $\left[v_{min}^{\prime },v_{max}^{\prime }\right]$, where the maximum feasible speed limit $v_{max}^{\prime }$ is considered to be the coordinated optimal speed of these two adjacent intersections.

Nonetheless, the absence of available vehicle speed in the set above doesn’t imply an obligatory stop at the stop line. Instead, it merely signals the impracticality of traversing both the *i*th and *i* + 1 th intersections at a consistent speed. This consideration aligns with the dynamic nature of traffic scenarios, where variables such as traffic density, signal timings, and potential congestion impact the feasibility of maintaining a constant speed between consecutive intersections.

In this situation, the speed of the *i*th intersection and *i +* 1 th intersection will be optimized separately using the method of vehicle trajectory optimization for single intersection.

### 3.3. Joint Control of Traffic Signal and Vehicle Speed Optimization

To assess the comparative effectiveness of integrating vehicle speed control with traffic signal control against using each method independently, four distinct scenarios were incorporated in the comprehensive case study of the entire network:

1—The current situation (without signal control or vehicle speed control);

2—The proposed signal control method which optimizes the parameter of signal timings and signal offsets (signal control only);

3—The proposed vehicle control method which is based on the current signal control plan (vehicle speed control only);

4—The proposed vehicle control method which is based on the optimal signal control plan found by the proposed signal control method (the combination of signal control and vehicle speed control).

**Figure 7:**
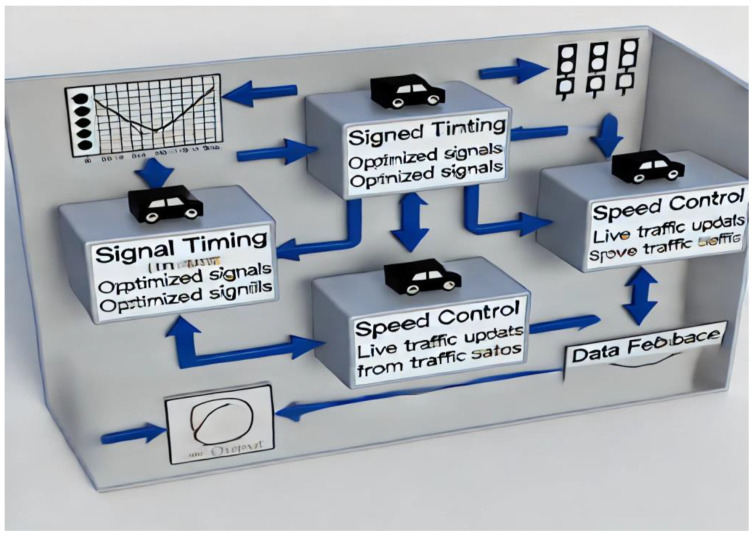
Interaction between signal timing, speed control, and real-time data feedback for traffic efficiency.

This section delves into the synergy achieved by combining vehicle speed control and traffic signal control strategies, taking into consideration factors such as signal timings, traffic density, and the interplay between connected and non-connected vehicles. Understanding how these methods interact in varying scenarios is crucial for optimizing overall traffic performance. By exploring the combined impact of these control strategies, this study aimed to provide insights into the potential benefits associated with integrating vehicle speed control and traffic signal control for enhanced traffic management. Figure 7 depicts the interaction between signal timing, speed control, and real-time data feedback for traffic efficiency.

### 3.4. Optimization of the Main and Side Roads

#### 3.4.1. Natural Advantages of Main Road Optimization

In traffic signal coordination, the main road is typically the most heavily trafficked route, especially in urban areas. Main roads often connect critical urban nodes, so optimizing the GreenWave bandwidth can help minimize travel time and the number of stops, thereby increasing the throughput of vehicles.

- The Role of GreenWave Optimization: Optimizing the GreenWave bandwidth means that vehicles on the main road can pass through multiple intersections continuously without encountering red lights, provided they travel within a certain speed range. This reduces the number of stops and delays on the main road, thereby lowering fuel consumption and CO_2_ emissions.
- Prioritizing Main Road Traffic: Since traffic volumes on the main road are generally higher than on side roads, prioritizing signal optimization for the main road helps improve the overall efficiency of the traffic system.

#### 3.4.2. Balanced Signal Optimization Strategies between Main Roads and Side Roads

Signal coordination systems need to consider multi-dimensional optimization strategies. These strategies should balance the needs of both the main road and side roads without significantly sacrificing the traffic flow on either.

- Dynamic Signal Adjustment: Signal timing can be adjusted based on real-time traffic flow, prioritizing the main road during peak hours while shortening green light times or increasing green light duration for side roads during off-peak hours. This ensures that side roads have opportunities to merge onto the main road without causing excessive congestion.
- Optimization of Signal Cycle Allocation: To avoid excessive queuing on side roads, the system can set a minimum green light duration for side roads within each signal cycle. This ensures that even when the main road has high traffic volumes, side roads still receive a minimal amount of green light time and are not subjected to excessively long red lights.
- Segmented Optimization and Coordination: For more refined optimization, the signal system could be divided into multiple segments. The main road and side roads could be optimized independently in different segments, while dynamic adjustments are made at intersection points through signal coordination. This approach ensures smooth traffic on the main road while providing reasonable access for side road vehicles.

#### 3.4.3. Further Research and Applications

Future research should explore how emerging technologies (e.g., artificial intelligence, machine learning) can enable smarter signal coordination strategies to better address the trade-offs between the main road and side roads. By analyzing real-time traffic data and incorporating traffic models, signal control systems can become more adaptive and responsive, maximizing the overall efficiency of traffic flow.

## 4. Case Study

### 4.1. Simulation Area

**Figure 8:**
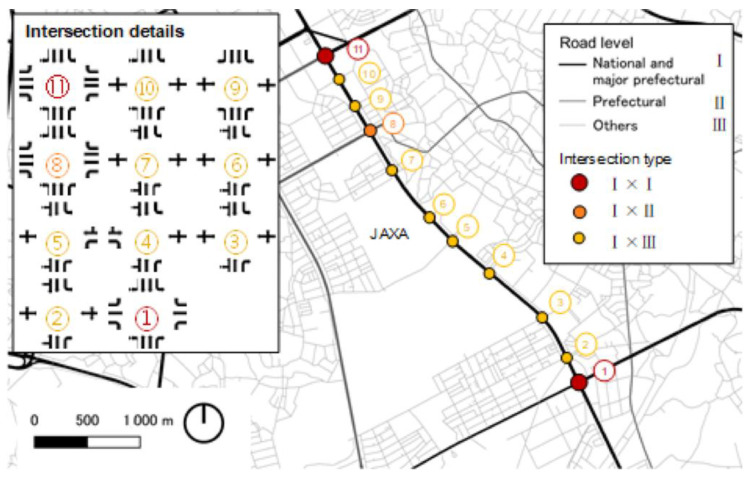
Simulation area: Gakuen Higashiōdōri in Tsukuba, Ibaraki, Japan.

The effectiveness of the developed signal control model is evaluated through simulation using Vissim 8 and Visual Basic 2015 (VB 14.0). The optimization function is implemented in Visual Basic, and the solution is seamlessly integrated with the Vissim 8 software using the COM interface. Notably, the simulation environment is configured using real-world vehicle data, allowing for the adjustment of Vissim 8 parameters to simulate realistic scenarios. As depicted in Figure 8, the study area encompasses an arterial road featuring eleven interconnected intersections along Gakuen Higashiōdōri in Tsukuba, Ibaraki, Japan.

- Intersection Numbering: The numbers correspond to the intersection IDs, which are labeled from 1 to 11, likely in sequence along the arterial road. It may help to mention how these intersections are arranged or the significance of their numbering.
- Intersection Type: The colors (red, orange, yellow) represent different types of intersections (e.g., I × I, I × II, I × III), which are already explained in the figure legend. If further clarification is required, you can explain the different intersection types in the text (e.g., major vs. minor intersections, or intersections between different road levels).
- Road Levels: The Roman numerals (I, II, III) correspond to different levels of roads (National, Prefectural, and Others), which are explained in the legend. You might want to expand on this in the text to explain how these different road levels interact in the network.

#### 4.1.1. Traffic Volume and Signal Settings

Traffic volume and signal scheme data for the simulation area were gathered through a field survey conducted during the evening peak, with the data collection period spanning one hour. The survey data of the current traffic volume are shown in Table 2. The traffic volume on the arterial road during the evening peak is around 1200 veh/h, while the traffic volume during the morning peak is around 1800 veh/h and traffic volume during 12 o’clock is around 900 veh/h.

The trajectory of vehicles on the north arterial and the south arterial road are both collected by Vissim to evaluate the effect of the signal control model. The vehicle composition of each lane consists of 98% cars and 2% of buses.

The proposed signal control model is based on the current signal control scheme during the evening peak. The signal timing data is also collected by the field survey during the evening peak. The cycle length of the original traffic signal control plan of each intersection is the same value. All the cycle lengths are 140 s.

#### 4.1.2. Simulation Progress

**Figure 9:**
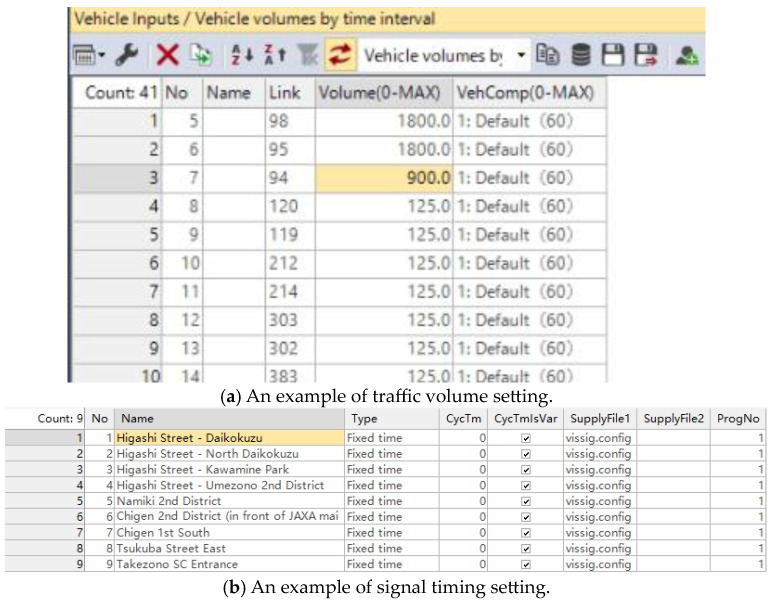
Basic data settings in Vissim.

Firstly, the investigated evening peak traffic volume data and signal control plan needs to be inputted into the Vissim software, as shown in Figure 9. The optimal signal timing for each intersection is calculated by out external signal control program. The obtained results are then inputted into Vissim software through the interface for simulation experiment, and the simulation results are used to evaluate the quality of different signal control schemes.

**Figure 10:**
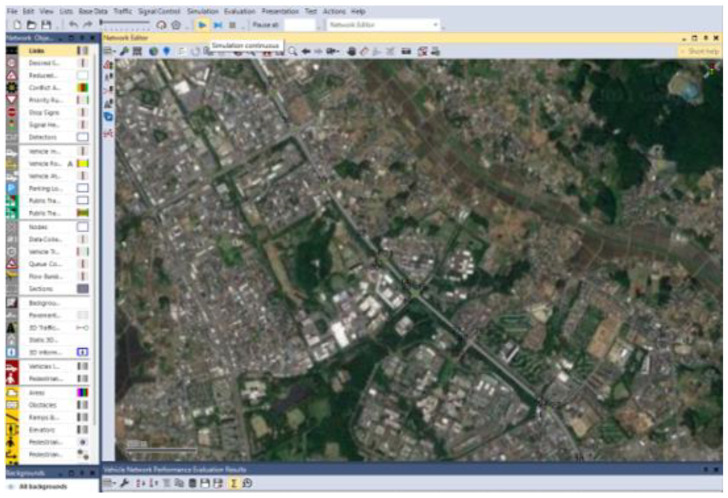
Simulation evaluation in Vissim.

Secondly, the interface, which is shown is Figure 10, is used to conduct simulation experiments with the Vissim software, and the obtained simulation evaluation results of the road network will appear at the bottom of the interface. By sorting and analyzing these simulation data, the simulation conclusions obtained will be presented in the following sections. Based on the figure, it appears that the numbers on the map are likely intersection and link IDs, which could represent locations along a traffic network used for simulation or evaluation purposes.

### 4.2. Solution Technique

#### 4.2.1. Scenario 1: Comparison of the Simulation Results with Different Speed Control Methods

To validate the viability of the proposed vehicle speed control model, we opt to simulate the single-intersection speed control model as a benchmark for comparative analysis. This approach ensures a robust assessment of the proposed model’s efficacy. In the simulation, key performance indicators such as delay, the count of vehicle stops, fuel consumption, and CO_2_ emissions are chosen as evaluation metrics to gauge the optimal impact comprehensively. This selection aligns with contemporary research methodologies in traffic engineering, focusing on a holistic evaluation of traffic control interventions.

Expanding our understanding, the chosen data analysis area spans the road section from intersection 1 to intersection 11, encompassing a real-world stretch of Gakuen Higashiōdōri in Tsukuba, Ibaraki, Japan. This particular section provides a representative snapshot of the overall performance of the vehicle speed control model in a complex urban context. Importantly, vehicles traverse this road section from intersection 1 to intersection 11, capturing the dynamic interactions and outcomes of the proposed model across multiple signalized intersections.

**Figure 11:**
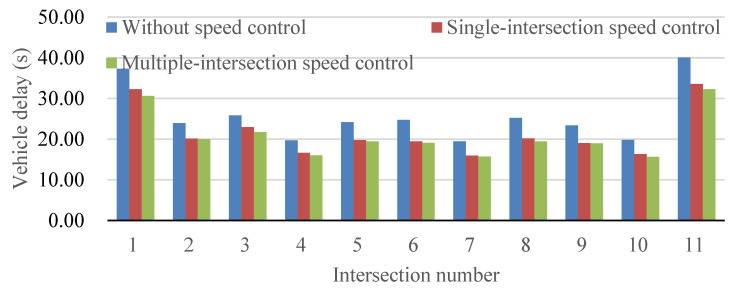
Comparison of average vehicle delay with different speed control methods.

Figure 11 illustrates a comparative analysis of average vehicle delay across distinct scenarios, including the current state (without speed control), a conventional single-intersection speed control method, and the proposed speed control method, which integrates speed control across multiple intersections. This approach aligns with contemporary research trends, recognizing the importance of evaluating traffic interventions in diverse settings for robust applicability. Examining the outcomes, it is evident that the proposed speed control method significantly reduces average vehicle delay compared to both the current situation and the conventional single-intersection speed control method. This improvement is attributed to the minimized stops at intersections, showcasing the model’s effectiveness in optimizing traffic flow dynamics. These findings emphasize the positive impact of the proposed speed control method on enhancing the traffic capacity of the arterial road and maximizing the utilization of green signal time. To enhance our comprehension, it is essential to delve into the nuanced aspects of traffic optimization. The minimal difference, within 5%, between the maximum and minimum reduction in average vehicle delay suggests that the efficacy of the proposed speed control method remains consistent across various intersections. This insight underscores the adaptability and reliability of the model, irrespective of the specific location of the intersection, reinforcing its potential for widespread applicability in diverse urban contexts.

**Figure 12:**
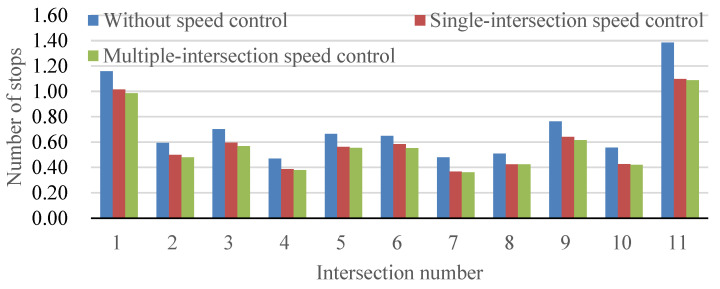
Comparison of the number of vehicles stops with different speed control methods.

Figure 12 displays a comparative analysis of the number of vehicle stops across different scenarios, including the current state (without speed control), a conventional single-intersection speed control method, and the proposed speed control method, which integrates speed control across multiple intersections. This comparative approach aligns with contemporary research methodologies, emphasizing a comprehensive evaluation of traffic interventions in varied settings. Analyzing the results, it is evident that the proposed speed control method significantly reduces the number of vehicle stops when compared to both the current situation and the conventional single-intersection speed control method. This reduction stems from the model’s effectiveness in minimizing stops at intersections, demonstrating its impact on improving overall traffic flow dynamics. These findings emphasize the positive influence of the proposed speed control method on enhancing the traffic capacity of the arterial road and optimizing the efficient use of green signal time. To deepen our understanding, it is pertinent to explore the nuanced aspects of traffic optimization. The minimal difference, within 5%, between the maximum and minimum reduction in the number of vehicle stops reinforces the consistency of the proposed speed control method’s optimization effect across various intersections. This observation underscores the model’s adaptability and reliability, irrespective of the specific location of the intersection, bolstering its potential for widespread applicability in diverse urban scenarios.

#### 4.2.2. Scenario 2: Comparison of Simulation Results with Different Speed Control Methods

**Figure 13:**
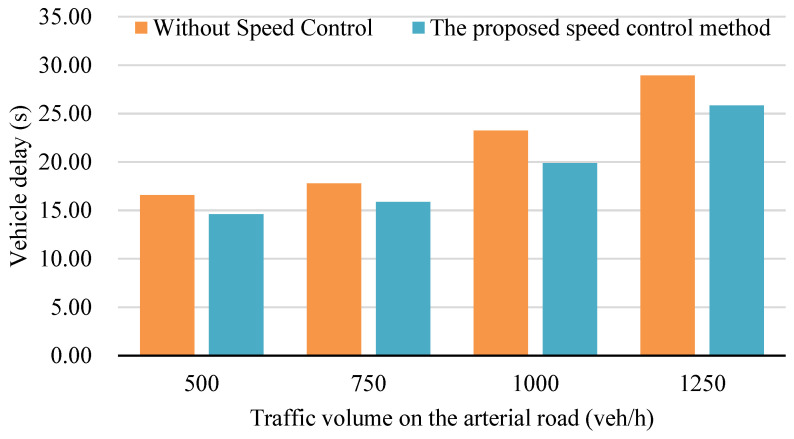
Comparison of average vehicle delay at different traffic volumes.

Figure 13 presents a comparative assessment of average vehicle delay across four distinct scenarios, each reflecting different traffic flow densities. The results demonstrate a noteworthy reduction in average vehicle delay when employing the speed control method in a connected vehicle environment, ranging from approximately 10% to 24%. This reduction is particularly pronounced in situations characterized by medium traffic flow density, specifically at a volume of 1000 vehicles per hour. Understanding these findings requires an exploration of the role of reduced sharp accelerations or decelerations, aligning with principles from traffic engineering that advocate for smoother vehicle flow, especially in moderate traffic conditions.

Delving into the details, it is essential to consider the implications of traffic flow density on the effectiveness of the proposed speed control method. Beyond a traffic volume of 1000 vehicles per hour, the efficacy of the method diminishes. This decline is attributed to the heightened difficulty for vehicles to adjust their speed in densely congested scenarios, where maneuverability becomes challenging. Consequently, the reduction in average vehicle delay becomes less conspicuous under high traffic flow density conditions compared to scenarios with medium traffic flow density. This nuanced understanding aligns with the intricacies of real-world traffic situations, highlighting the variable impact of speed control methods in different traffic conditions.

**Figure 14:**
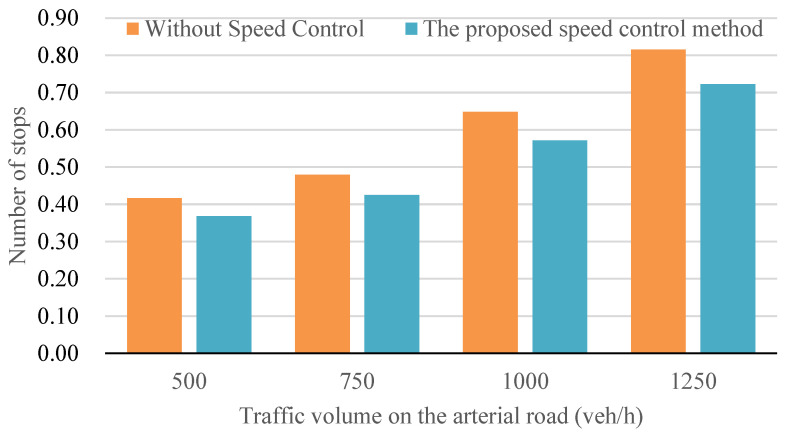
Comparison of the number of vehicles stop at different traffic volumes.

Figure 14 illustrates a comparative analysis of the number of vehicle stops across four scenarios, each corresponding to varying traffic flow densities. The results reveal a reduction in vehicle stops ranging from approximately 10% to 24% when implementing the speed control method in a connected environment. This reduction is especially pronounced in scenarios marked by medium traffic flow density, specifically at a traffic volume of 1000 vehicles per hour. Understanding the significance of this reduction involves recognizing the role of connected vehicle systems, designed to minimize disruptions by smoothing accelerations or decelerations through real-time communication and control. Expanding on this, it is crucial to delve into the dynamics of connected vehicle systems. These systems leverage advanced technologies, including the connected vehicle communication and predictive analytics, to enhance traffic flow efficiency. The reduction in vehicle stops aligns with the broader goals of connected vehicle systems, illustrating their capacity to create a more seamless and responsive traffic environment, particularly in conditions of moderate congestion.

However, as traffic flow density increases to high levels, exceeding 1000 vehicles per hour, the efficacy of the proposed speed control method diminishes. This challenge arises from the heightened difficulty for vehicles to adjust speed in densely congested situations, where maneuverability is constrained. Consequently, the reduction in vehicle stops becomes less apparent under high traffic flow density conditions compared to scenarios with medium traffic flow density. This nuanced observation underscores the contextual influence of speed control methods in diverse traffic scenarios, recognizing the dynamic nature of urban mobility challenges.

#### 4.2.3. Scenario 3: Comparison of Simulation Results with Different Penetration Rates of Connected Vehicles

To assess the efficacy of the suggested speed control algorithm across various penetration rates of connected vehicles, we diversified the penetration rate across a range from 0% to 100%. The case study encompassed eleven distinct scenarios, providing a comprehensive evaluation of the algorithm’s performance throughout the entire network:

1—0% of the vehicles are connected vehicles;

2—10% of the vehicles are connected vehicles;

3—20% of the vehicles are connected vehicles;

4—30% of the vehicles are connected vehicles;

5—40% of the vehicles are connected vehicles;

6—50% of the vehicles are connected vehicles;

7—60% of the vehicles are connected vehicles;

8—70% of the vehicles are connected vehicles;

9—80% of the vehicles are connected vehicles;

10—90% of the vehicles are connected vehicles;

11—100% of the vehicles are connected vehicles.

Introducing variability in the penetration rate allows for a nuanced understanding of how the proposed algorithm adapts to different levels of connected vehicle penetration rate. This section considers factors, for example the interplay between connected and non-connected vehicles, enriching the analysis of the algorithm’s effectiveness in diverse traffic conditions.

**Figure 15:**
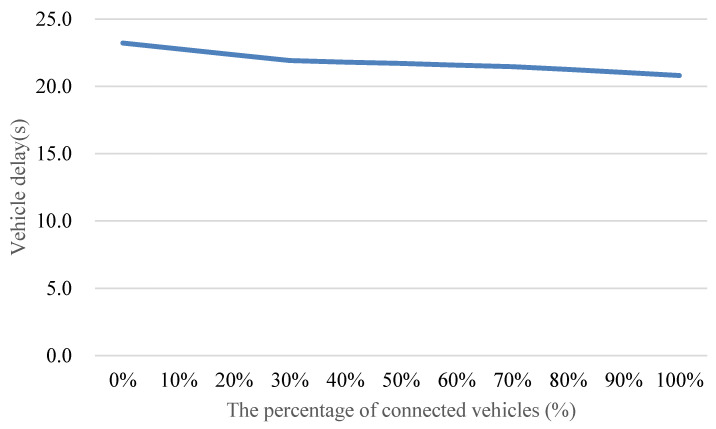
Comparison of average vehicle delay with different penetration rates of connected vehicles.

Figure 15 demonstrates that by implementing the proposed method, the reduction in average vehicle delay increases proportionally with the penetration rate of connected vehicles, ranging from 0% to 100%. The observed reduction in average vehicle delay reaches a peak of approximately 24% with a full adoption of connected vehicles. Expanding our understanding, it is important to note that this reduction signifies the efficiency gains achieved through enhanced communication and coordination among vehicles in a connected environment. Additionally, even at a relatively modest penetration rate of connected vehicles, such as 30%, the proposed method still delivers a meaningful reduction in average vehicle delay, around 10%. This underscores the adaptability and effectiveness of the method in influencing traffic dynamics, even with a partial integration of connected vehicles. Understanding the dynamics of partial integration is crucial for real-world scenarios where the transition to a fully connected vehicle fleet may take time. To delve further into the underlying principles, it is essential to recognize that achieving effective speed guidance necessitates a penetration rate exceeding 30% of connected vehicles. This requirement stems from the increased prominence of the car-following phenomenon when the penetration rate surpasses 30%. In such instances, the formation of vehicle platoons becomes more pronounced, facilitating more vehicles to attain optimal speeds. This insight contributes to our understanding of the threshold for realizing the full benefits of connected vehicle technologies in optimizing traffic flow.

**Figure 16:**
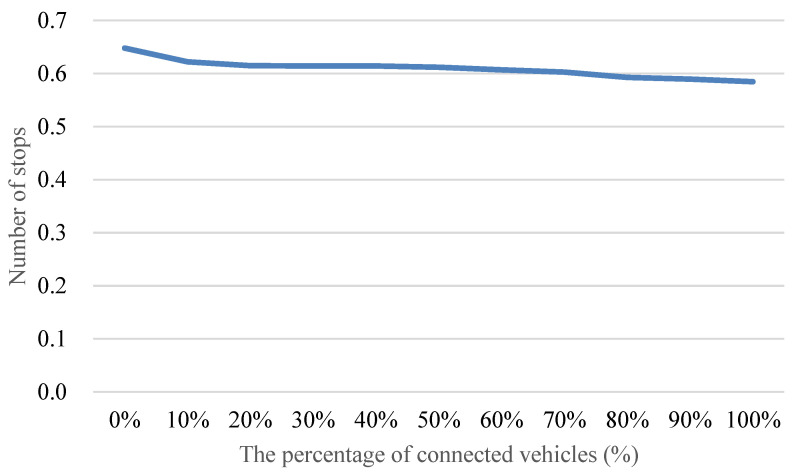
Comparison of the number of vehicle stops with different penetration rates of connected vehicles.

Figure 16 demonstrates that implementing the proposed method leads to an improvement in the reduction of the number of vehicle stops as the penetration rate of connected vehicles varies from 0% to 100%. The observed reduction in the number of vehicle stops peaks at approximately 24% with full adoption of connected vehicles. It is crucial to explore the underlying mechanisms driving this reduction, acknowledging the pivotal role of connected vehicle technologies in mitigating disruptions and optimizing traffic flow. Furthermore, even at a lower penetration rate of connected vehicles, such as 30%, the proposed method still yields a significant reduction in the number of vehicle stops, around 10%. This emphasizes the method’s effectiveness in influencing traffic patterns even in scenarios where the integration of connected vehicles is only partial. Recognizing the impact of partial integration is essential for practical implementation, considering the gradual adoption of connected vehicle technologies. To delve deeper into the dynamics, it is essential to recognize that achieving effective speed guidance requires a penetration rate exceeding 30% of connected vehicles. This requirement is attributed to the increased prominence of the car following phenomenon when the penetration rate surpasses 30%. In such scenarios, vehicles may form platoons, facilitating more vehicles to reach optimal speeds. Understanding these dynamics enhances our insight into the conditions necessary for maximizing the benefits of connected vehicle technologies in optimizing traffic flow.

#### 4.2.4. Scenario 4: Comparison of Simulation Results with Control Methods on Each Intersection

To assess whether the combined vehicle speed control and signal control method outperforms using either method individually, four comparative experiments are conducted within the same traffic network. These experiments aim to evaluate the effectiveness of different control strategies at each intersection, considering the dynamic interaction between signal and vehicle speed optimization.

In the simulation experiments, key performance metrics such as delay, number of vehicle stops, fuel consumption, and CO_2_ emissions are selected as evaluation indexes to comprehensively assess the impact of the control methods. The chosen data analysis area spans the road section from intersection 1 to intersection 11, simulating the vehicles’ travel trajectory along this route. This experimental setup aligns with real-world considerations, providing a comprehensive evaluation of the joint control model’s ability to enhance traffic performance across a network of connected intersections. The selection of diverse evaluation metrics ensures a multifaceted assessment, capturing various aspects of traffic efficiency and environmental impact.

**Figure 17:**
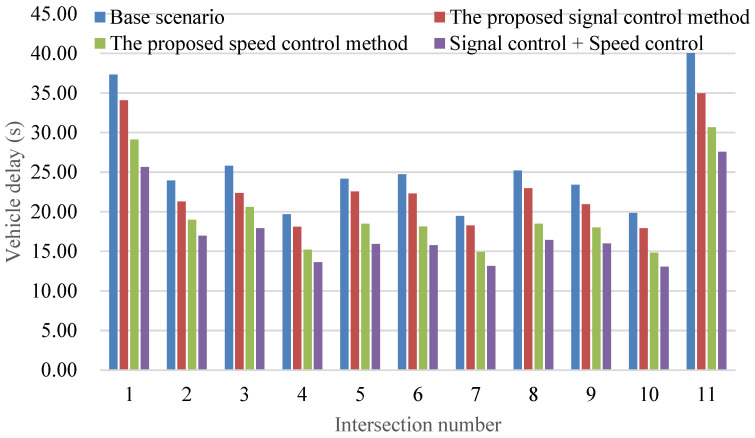
Comparison of vehicle delay at intersections under different control methods.

Figure 17 presents a comprehensive analysis of average vehicle delay across various scenarios, including the baseline condition (no speed control), the proposed signal control method optimizing signal timings and offsets (signal control only), the proposed vehicle control method utilizing the current signal control plan (vehicle speed control only), and the integration of the proposed vehicle control method with the optimal signal control plan identified through the proposed signal control method (combined signal control and vehicle speed control). This extensive investigation goes beyond traditional traffic signal and vehicle speed control methods by integrating principles from dynamic vehicle speed control, a distinguishing feature of the proposed approach known for its substantial reduction in average vehicle delay. This reduction not only emphasizes the positive impact on traffic capacity but also highlights the efficient utilization of green signal time.

The narrow range (within 5%) between the maximum and minimum reductions in average vehicle delay underscores the robustness of the proposed joint control method. This consistent performance implies that the method’s effectiveness remains relatively unaffected by specific spatial configurations of intersections. This observation emphasizes the versatility and broad applicability of the integrated approach across diverse urban traffic scenarios, showcasing its adaptability to varying intersection layouts and traffic conditions.

#### 4.2.5. Scenario 5: Comparison of Simulation Results with Different Traffic Volumes

To verify whether the performance of the combination of vehicle speed control and traffic signal control is better than simply using the signal control method or simply using the vehicle speed control method, we conducted four comparison experiments based on the same traffic network and evaluate the performance of different control methods under different traffic volume.

**Figure 18:**
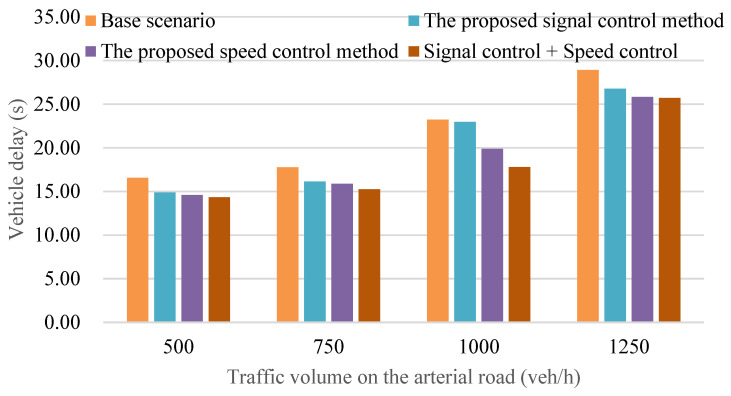
Comparison of average vehicle delay with different traffic volumes.

Figure 18 illustrates a comparative analysis of average vehicle delay across four distinct cases, each representing varying traffic flow densities. The findings from Figure 18 reveal that the implementation of the joint control method of traffic signal and vehicle speed under a connected environment results in a noteworthy reduction in average vehicle delay, ranging from approximately 10% to 35%. This reduction is particularly significant in situations characterized by medium traffic flow density, specifically at a traffic volume of 1000 vehicles per hour. The improvement observed can be attributed to the mitigation of sharp accelerations or decelerations during the driving process, a key aspect of the joint control method’s functionality.

It is essential to recognize that the effectiveness of the joint control method is subject to the traffic flow density, as observed in the results. In scenarios where traffic flow density reaches high levels, exceeding 1000 vehicles per hour, the ability of vehicles to adjust their speed becomes more challenging. Consequently, the reduction in average vehicle delay becomes less pronounced under high traffic flow density conditions compared to scenarios with medium traffic flow density. This observation aligns with real-world traffic dynamics, emphasizing the nuanced impact of the joint control method in diverse traffic scenarios.

#### 4.2.6. Scenario 6: Comparison of Simulation Results with Different Penetration Rate of Connected Vehicles

To assess the efficacy of the joint control algorithm across diverse penetration rates of connected vehicles, we introduced variability in the penetration rate, ranging from 0% to 100%. The case study incorporated eleven distinct scenarios, providing a comprehensive evaluation of the algorithm’s performance across the entire network:

1—0% of the vehicles are connected vehicles;

2—10% of the vehicles are connected vehicles;

3—20% of the vehicles are connected vehicles;

4—30% of the vehicles are connected vehicles;

5—40% of the vehicles are connected vehicles;

6—50% of the vehicles are connected vehicles;

7—60% of the vehicles are connected vehicles;

8—70% of the vehicles are connected vehicles;

9—80% of the vehicles are connected vehicles;

10—90% of the vehicles are connected vehicles;

11—100% of the vehicles are connected vehicles.

Introducing variability in the penetration rate allows for a nuanced understanding of how the joint control algorithm adapts to different levels of connected vehicle penetration rate. This examination considers factors, for example the interplay between connected and non-connected vehicles, enriching the analysis of the algorithm’s effectiveness in diverse traffic conditions.

**Figure 19:**
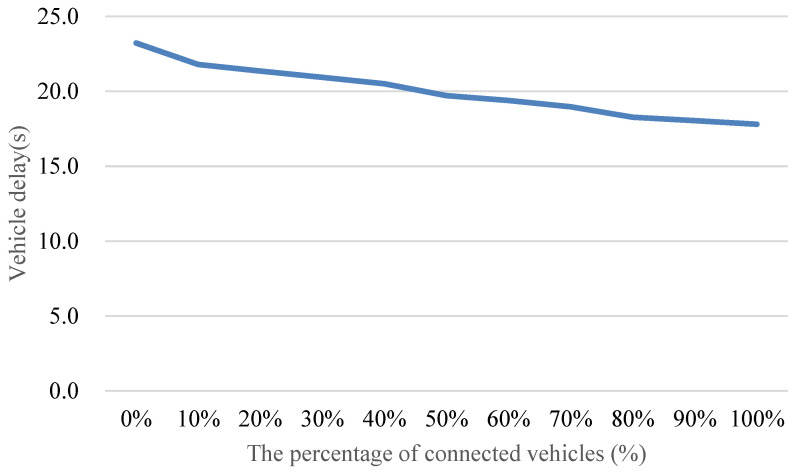
Comparison of average vehicle delay with different penetration rates of connected vehicles.

Figure 19 illustrates the impact of the proposed method on average vehicle delay as the penetration rate of connected vehicles varies from 0% to 100%. The findings reveal a substantial reduction in average vehicle delay, reaching up to approximately 35% with the full adoption of connected vehicles. Notably, even at a relatively modest penetration rate, such as 30%, the proposed method can still achieve a noteworthy reduction of around 10% in average vehicle delay.

Understanding the dynamics of connected vehicle penetration is crucial for interpreting these results. As the penetration rate increases beyond 30%, the car following phenomenon becomes more prominent. This phenomenon is characterized by vehicles forming platoons, enhancing the collective ability to reach and maintain optimal speeds. Consequently, a penetration rate exceeding 30% becomes essential for effectively guiding vehicle speeds and achieving the full benefits of the proposed method. This observation aligns with the principles of connected vehicle systems, emphasizing the importance of network-wide adoption for optimal performance.

## 5. Results and Discussion

### 5.1. Vehicle Trajectory Comparison Diagram

**Figure 20:**
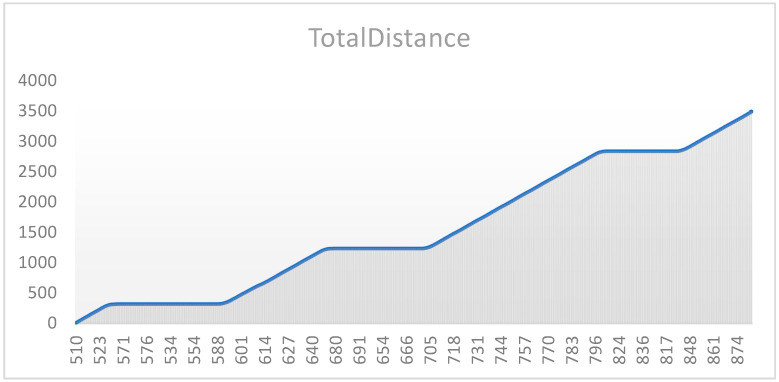
Vehicle trajectories at each intersection before speed and signal optimization control.

According to the intersection information of the known map and the information of vehicles in the network of vehicles, the vehicle track data before optimization are shown in Figure 20.

**Figure 21:**
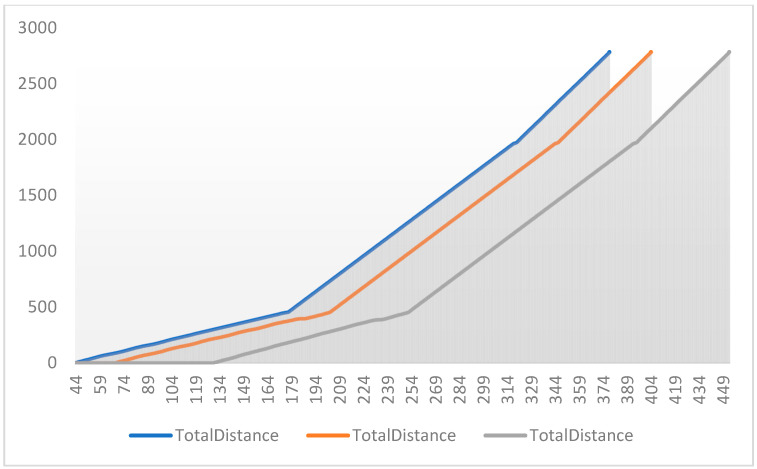
Vehicle trajectory diagram with the speed Strategy for a single intersection.

The following shows the total distance traveled by the vehicle trajectory under speed optimization control at a single intersection based on different timestamps. The vehicle trajectory diagram under optimized speed control is presented in Figure 21.

**Figure 22:**
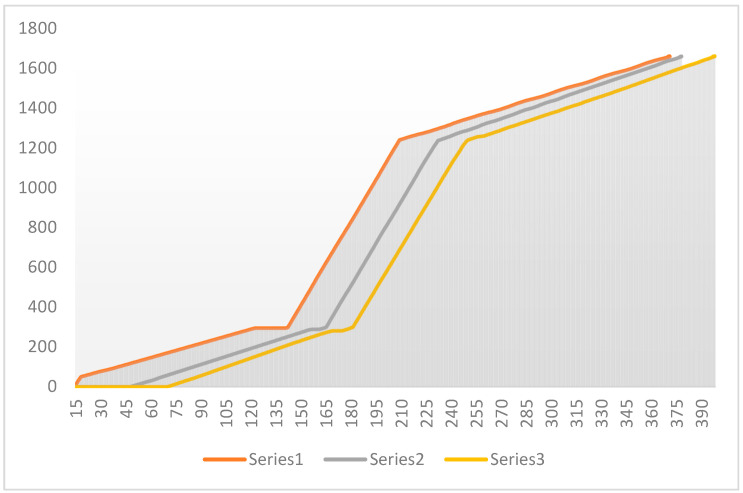
Vehicle trajectory diagram with the speed strategy for multiple intersections.

The vehicle trajectory with the speed strategy at multiple intersections based on different timestamps is shown in Figure 22.

Point ①: This is a point where several series converge, suggesting that all traffic flows were similarly affected by signal control at this time, likely due to red lights or signal coordination, causing the driving conditions of different traffic flows to become uniform.

Point ②: At this point, the blue series (Series 5) significantly diverges from the others and shows a sharp increase in speed, possibly reflecting a green light after signal coordination, allowing this traffic flow to pass quickly through several intersections. The remaining series recover their speed more slowly, suggesting that they might have experienced longer signal control delays.

**Figure 23:**
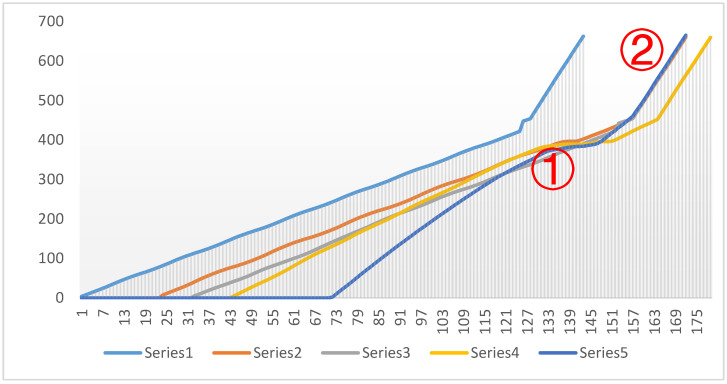
Trajectory diagram of traffic signal coordination between the main road and side roads.

Effectiveness of Signal Coordination: The chart shows that after point ①, the blue series gained a significant speed advantage, indicating that the signal coordination system prioritized traffic flow on the main road. However, other series recovered more slowly after point ②, which could mean that signal control on side roads was not well synchronized with the main road. The specific details are illustrated in Figure 23.

Based on the above charts, not only are the vehicle trajectories compared with and without speed control, but the comparison between speed control strategies for multiple intersections and a single intersection is also presented. These charts clearly illustrate the differences in traffic flow dynamics and highlight the advantages of the proposed speed control method.

### 5.2. Comparison of the Simulation Results

**Figure 24:**
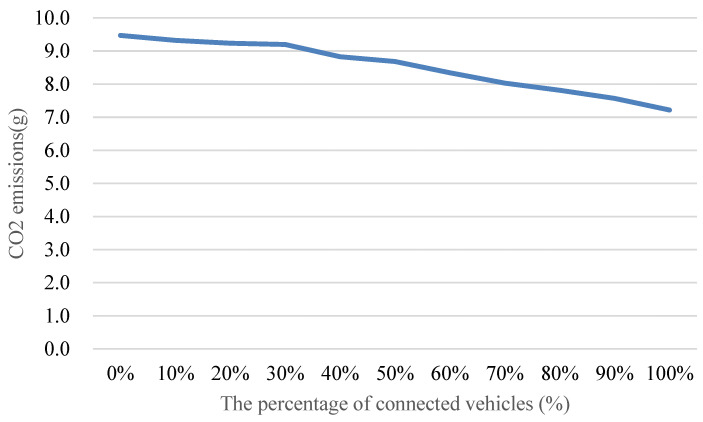
Comparison of the CO_2_ emissions with different penetration rates of connected vehicles.

Analyzing simulation results unveils the remarkable efficacy of the proposed vehicle speed control method, showcasing substantial reductions in vehicle delay, the number of stops, fuel consumption, and CO_2_ emissions. These outcomes contribute to the ongoing discourse on sustainable urban mobility, aligning with global efforts to minimize the environmental impact of transportation systems. Furthermore, the method’s superior performance when optimizing vehicle speed for two adjacent intersections simultaneously highlights its potential to enhance traffic flow across connected intersections. This aligns with the growing emphasis on connected and automated vehicle technologies in shaping the future of transportation networks, as shown in Figure 24.

**Figure 25:**
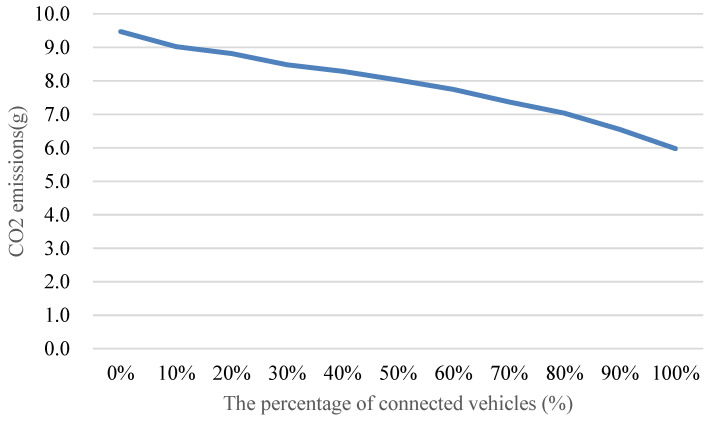
Comparison of the CO_2_ emissions with different penetration rates of connected vehicles.

The speed control system then determines the optimal speed based on the current signal control plan. To provide a comprehensive evaluation, the performance of employing only the vehicle speed control method is examined. Subsequently, to explore the synergies between vehicle speed control and signal control, the optimal speed based on the signal control plan derived from the proposed signal control method is calculated. This multi-faceted approach aims to investigate the collective impact of joint optimization on traffic signal and vehicle speed, as shown in Figure 25.

## 6. Conclusions

By integrating signal control with vehicle speed control, the innovative method proposed in this paper demonstrates significant advantages in improving the efficiency of traffic networks, reducing delays and stops, and lowering energy consumption and emissions. The simulation results indicate that the introduction of connected vehicle technology allows signal control systems to better respond to dynamic traffic conditions, with the optimization effects becoming more pronounced as the penetration rate of connected vehicles increases. Future research can focus on verifying the effectiveness of this method in real-world traffic environments and further optimizing the model. Moreover, with the rapid development of autonomous driving technologies, integrating these new technologies with existing signal control and vehicle speed optimization algorithms may further enhance the intelligence and sustainability of traffic systems.

(1) Real-World Application Verification: Although the study achieved promising results in a simulation environment, future work should involve testing the method in real urban traffic environments to further verify its feasibility and effectiveness.
(2) Integration with Smart Transportation Technologies: As autonomous driving and artificial intelligence technologies continue to develop, future research could explore how to combine these emerging technologies with existing traffic signal and vehicle speed optimization methods to further enhance intelligent traffic management.
(3) Extension to Complex Traffic Networks: Future studies could apply this method to more complex traffic networks, including various types of roads, intersections, and different traffic patterns, to further expand its applicability.

In summary, this research demonstrates that by combining connected vehicle technology with signal control and vehicle speed optimization, it is possible to significantly improve traffic system efficiency, reduce delays and environmental impacts, and provide important insights and directions for future urban traffic management.

## Figures and Tables

**Table 1:** Notations and descriptions.

Parameter	Description	Unit
*g*	The green time of each signal phase	s
$D\left(g\right)$	The total travel delay of all vehicles	s
$D_{m}\left(g\right)$	The travel delay of each vehicle	s
*m*	The vehicle number	
*N*	The total number of vehicles that have been in the intersection of each signal phase	

**Table 2:** Traffic volume of each intersection (evening peak).

Intersection Number		North Entrance (veh/h)			South Entrance (veh/h)			West Entrance (veh/h)			East Entrance (veh/h)	
	Left	Straight	Right	Left	Straight	Right	Left	Straight	Right	Left	Straight	Right
11	144	1251	89	215	1265	90	213	1254	150	187	1298	89
10	57	1247	58	48	1206	63	54	52	59	62	137	56
9	100	1208	68	86	1203	119	52	50	91	137	170	137
8	68	1287	85	82	1294	91	98	141	493	102	224	159
7	49	1283	52	47	1220	157	57	69	52	74	61	61
6	149	1265	142	73	1206	59	116	0	202	100	0	60
5	69	1260	64	58	1228	50	160	48	56	37	40	50
4	193	1286	70	100	1280	116	89	94	76	70	76	317
3	43	1298	168	108	1284	270	205	130	62	167	262	113
2	54	1290	103	100	1275	71	44	90	164	56	48	60
1	264	1281	128	241	1228	185	269	1217	211	230	1294	202

## Data Availability

Data are unavailable due to privacy or ethical restrictions.
